# Three years of pandemic stress and staffing challenges: a retrospective qualitative study of COVID-19 impacts on frontline healthcare workers’ mental health and wellbeing

**DOI:** 10.1186/s12888-025-07348-4

**Published:** 2025-10-30

**Authors:** Brian En Chyi Lee, Mathew Ling, Leanne Boyd, Craig A. Olsson, Hannah Michelle LaFontaine Harvey, Jade Sheen

**Affiliations:** 1 SEED-Lifespan (Clinical Mental Health Care), School of Psychology, Faculty of Health, Deakin University, Burwood, VIC Australia; 2 Neami National, Melbourne, VIC Australia; 3 Eastern Health, Boxhill, VIC Australia; 4 Centre for Adolescent Health, Murdoch Children’s Research Institute, Royal Children’s Hospital, Melbourne, VIC Australia; 5 Department of Paediatrics, University of Melbourne, Parkvile, VIC Australia

**Keywords:** Healthcare, COVID, Mental Health, Anxiety, Work-Family

## Abstract

**Background:**

Healthcare systems have faced unprecedented demand over the course of the COVID-19 pandemic, placing significant burdens on frontline doctors, nurses, and support staff. Many studies have thus observed significant impacts on frontline healthcare workers’ mental health and wellbeing, however, few studies have explored the evolving nature of healthcare roles and its impacts on healthcare workers. This study retrospectively explored the changing nature of frontline healthcare roles over the last three years of the pandemic, and its impact on stressors and supports related to frontline healthcare workers’ mental health and wellbeing.

**Method:**

A total of 11 Victorian (Australia) frontline healthcare workers from frontline wards participated in retrospective semi-structured interviews between March 2023 to April 2023. Data was analysed using thematic analysis, using a reflective approach to identify patterns across participant narratives and experiences.

**Results:**

Three superordinate and nine subordinate themes were identified. Themes revolved around (1) COVID-19 anxiety and work related stressors in the initial and later stages of the pandemic, (2) The great resignation of healthcare workers that led to workforce issues, fatigue and exhaustion, which subsequently impacted team dynamics, workplace culture, engagement at work, and patient care, and (3) Strained social connections and work to family spillover effects during the COVID-19 era, as well as the key relational supports that helped healthcare workers manage stressors.

**Conclusion:**

The COVID-19 pandemic appears to have amplified existing challenges in healthcare provision and highlighted the psychological burden of healthcare roles on staff. Workplace demands have shifted from crisis management of acute risk of COVID-19 infections to now managing staffing shortages, presenting new challenges that require innovative solutions to ensure frontline HCWs’ mental health are protected. There is thus a need to adapt staff supports to manage the excessive demand on staff now, and the findings here offer recommendations for governments, health organisations, and healthcare leaders to design policies and supports that meets the needs of frontline HCW’s mental health and wellbeing.

**Supplementary Information:**

The online version contains supplementary material available at 10.1186/s12888-025-07348-4.

## Introduction

Over the last two decades, many have raised major concerns over the poor state of mental health and wellbeing among doctors, nurses, allied health, and support staff alike. There has been extensive evidence showing that severe burnout and depression have been consistently prevalent among healthcare workers (HCWs) [1–4], conditions some have suggested are associated with the high rates of suicide found among this cohort [5, 6]. Recently, research suggests that these mental health issues have substantially worsened and additional psychological challenges have been emerging, as the COVID-19 pandemic has led to a surge in workplace demands and stressors in the healthcare sector, placing unprecedented pressures on HCWs, particularly for those working in frontline roles [7, 8].

During the COVID-19 pandemic, many studies have shown that working in frontline roles have placed HCWs at significant risks of mental health issues [9–11]. In these studies, frontline HCWs are typically defined as those working in high-risk clinical environments such as emergency departments, intensive care units, COVID-19 wards, and aged care settings, where exposure to COVID-19 patients and infections was most prevalent. In the initial phase of the pandemic, when crisis management of infection outbreaks was the priority, sources of stress for frontline HCWs included managing challenges with the surge in patient care demands [12, 13], the pervasive uncertainty with COVID-19 outbreaks [14, 15], inconsistent protocols at work [13, 16], and heightened fears of being infected and infecting family members [17, 18]. Under these strained conditions, the toll on frontline HCWs’ mental health was extensive, with depression [9, 19], anxiety [9, 20], insomnia [21], and post-traumatic stress [22] widely evidenced as highly prevalent mental health issues among frontline HCWs during the initial phase of the pandemic.

Less is known about the mental health legacy, and the changing nature of workplace stress among frontline HCWs over the course of the COVID-19 pandemic. This gap is notable given the dynamic changes in healthcare roles during this period, as frontline HCWs faced the rapid and extensive spread of a novel virus [23], fluctuating infection control measures [24, 25], persistent staffing shortages [24, 26], and more complex patient care marked by the clinical management of a severe, unfamiliar disease [27–29]. However, while findings from the early stages of the pandemic have largely been consistent, by contrast, emerging research on frontline HCWs’ later experiences and mental health outcomes towards the end of the pandemic have been few and mixed. Some research have indicated a decrease in mental health issues with the height of the pandemic behind [30, 31], while other recent research have indicated prevailing mental health problems among frontline HCWs [10, 32, 33]. Current stressors underlying mental health problems in the frontline HCW workforce now is also unclear. Some studies have shown a decrease in primary pandemic stressors, including COVID-19 anxiety and traumatic stress related to disaster response [31, 34]. Other research, however, indicate an emergence of other workplace challenges and stressors triggered by COVID-19 impacts on the healthcare sector, including staff shortages [35, 36], increases in workplace violence [37] and work-family conflict [38].

Given these inconsistencies, the challenge now is to document and understand changes in stress among frontline HCWs, and to advise of the right types of mental health care services and supports, to ensure that the risk of mental health problems related to current demands and the lasting impact of pandemic on frontline HCWs are mitigated. It is clear that frontline HCWs remain vulnerable, underscoring the need to identify and explore past and present workplace stressors. Understanding the impacts of the pandemic on frontline HCWs’ work roles, both during the initial phase of the pandemic and presently, may facilitate improved mental health and wellbeing amongst the cohort through targeted prevention and intervention efforts. It is essential to capture the lived experiences of frontline HCWs to support the development of consumer-led supports and solutions, and to answer the calls for the workforce to directly inform key decision-making revolving their own outcomes [39].

This research explores frontline HCWs’ experiences of the changing nature in stressors and supports in their frontline roles over the last three years of the pandemic. Specifically, the aims were to investigate:

1. What were frontline HCWs’ experiences of working in frontline healthcare roles throughout the three years of the COVID-19 pandemic?
2. What were frontline HCWs’ reflections on their state of mental health and wellbeing throughout the three years of the COVID-19 pandemic?
3. According to frontline HCWs, what factors have helped support their mental health and wellbeing across the three years of the COVID-19 pandemic?

## Methods

Human ethics approval was provided by Deakin University High Risk Ethics Committee (HEAG 2020 − 296), Eastern Health’s Ethics Committee, and all partnering healthcare associations prior to commencement of this study. The report of this study is inline with the consolidated criteria for reporting of qualitative research [40] (COREQ; see Appendix A for further details).

### Recruitment and participants

This study is part of a longitudinal cohort mixed methods study on Australian frontline HCWs’ mental health and wellbeing. In the cohort study, frontline HCWs, including included doctors, nurses, allied health (e.g., social workers and pharmacists) and non-medical staff (e.g., personal care assistants and admin staff), participated in surveys across four timepoints. They were recruited from a state-wide health service (Eastern Health), and five major Australian healthcare associations: Australian medical association Vic (AMAVic), Australian Nursing and Midwifery Federation Vic (ANMFVic), Aged & Community Care Providers Association (ACCPA), Victorian Healthcare Association (VHA) and Health Services Union (HSU). Between March 2023 and April 2023, Victorian (Australia) frontline HCWs participating in the survey component of the cohort study, detailed further elsewhere [10], were invited to participate in semi-structured interviews. A final 11 frontline HCWs, three from the Emergency Department, three from Intensive Care Units, four from COVID-19 Wards, and one from aged care consented and participated in this qualitative study. Participants included nine nurses, one allied health and one non-medical staff. Nine were women and two were men. Five were below the age of 40 and six were 40 or above.

### Context

To aid the interpretation of this retrospective data analysis it is necessary to understand the context of frontline roles in Victoria during the COVID-19 pandemic. Victoria spent 185 days across six statewide lockdowns from March 2020 to October 2021. Lockdown restrictions included stay at home measures, a five-kilometre limit on traveling, mandatory use of masks and remote learning for children. Restrictions eased for the public by December 2021, however, many restrictions remained for frontline HCWs [41].

During January 2022, the Omicron wave led to the largest surge in hospitalisations and infections, triggering a code brown emergency in all hospitals [42], which meant non-essential health services halted, staff were to redeployed to critical units, and staff leave was cancelled [43].

At the time of data collection, all restrictions have eased, and the end of the COVID-19 pandemic emergency was declared by the Victorian Government and the WHO [44].

### Data collection procedure

Authors BL and HMLH conducted semi structured interviews that were recorded over the online platform Zoom, ranging from 41 min to 77 min (mean = 55 min, SD = 12 min). The interview included 10 questions on workplace demands, challenges during the pandemic, long-term career focus, mental health, wellbeing, coping strategies, and work-life balance (See Appendix B). Participants were asked to reflect on the three years of the COVID-19 pandemic retrospectively with key events being recognised, such as the delta wave, lockdowns, omicron wave and the code brown raised in Victorian hospitals. Interviews were then transcribed verbatim and sent to participants to review for accuracy and resonance with participants’ experiences. No participants made changes to the transcripts. Transcripts were then de-identified for data analysis.

### Data analysis procedure

This study employed reflexive thematic analysis, using NVIVO software to organise codes and emerging themes. The reflexive approach was chosen as it conceptualises themes as meaning based patterns, explicitly and conceptually, allowing the authors to contextualise lived experiences, and perceptions without aligning responses to a particular theoretical framework [45, 46]. This was important due to the unprecedented nature of the COVID-19 pandemic, making it a novel, complex and unique phenomenon.

Initially, BL immersed in the data through listening to interview recordings, reviewing interview notes, and reading transcripts. A preliminary inductive analysis was then conducted to generate micro level codes that reflected key experiences and reflections relevant to the research question that emerged from the data. These initial micro-level codes were then discussed with JS to determine patterns within the micro codes that formed the initial themes. BL then independently revisited the responses, finalising codes, identifying key quotes, and identifying recurring concepts and emerging themes. Consensus on final themes was finally reached through discussions with BL, JS and LB, and a final review of responses.

### Reflexivity

The analysis team (BL, JS, and LB) was made up of a diverse group of researchers that included a wide range of specialities and career stages. At the time of writing, BL was a PhD candidate and early career researcher in the field of social and relational psychology. JS was an Associate Professor in Clinical Psychology and family therapist, and LB was a Professor in Nursing, and Chief Nursing and Midwifery Officer in a large Victorian public healthcare network. As clinician researchers, both JS and LB were also working as healthcare workers during the COVID-19 pandemic and throughout the study period. LB was working in frontline nursing roles and JS, as an essential healthcare worker, provided clinical psychology services. All three authors were living and working in Victoria (Australia) throughout the COVID-19 pandemic and at the time of data collection and analysis.

Together the team brought together diverse perspectives from healthcare, public health, clinical psychology and relational psychology that shaped the paradigm and views of the team as a whole. These perspectives guided the process of identification and formulation of themes, which provided a comprehensive view of participants’ experiences with workplace stressors, mental health and wellbeing. To minimise bias and establish trustworthiness, BL maintained analytical journals, engaged in self-reflection, and participated in regular discussions with the broader data analysis team. Also, to ensure a robust analysis was employed, the themes were also closely reviewed against transcripts throughout the data analysis process, ensuring that themes aligned with participants’ experiences. Additionally, a team member outside of the data analysis team (HMLH) but who was still considerably involved in the interviews and reviewing transcripts, reviewed and checked themes to ensure they were consistent with participants’ experiences.

## Results

Overall, three superordinate themes were identified, with 9 subordinate themes (See Table 1). Within the results, quotes were italicised and presented alongside respective participant’s gender (F = Female, M = Male), age, occupation, and frontline units.

**Table 1:** Superordinate and subordinate themes

Superordinate Themes	Subordinate themes
The relentlessness of healthcare roles	COVID-19 Anxiety: Lived Experiences and Impacts Relentless demands and fatigue
The Great Resignation	Workforce gaps: The Impacts and Burden on Remaining Staff Disengaging and Leaving Patient care: The collateral impacts
Social connections in the COVID-19 era	Community disconnectedness Work to Family Spillover Effects: Impacts on Connections and Cohesion The Supportive Role of Workplace Relationships

### The relentlessness of healthcare roles

This theme relates to the cumulative and continuous stress healthcare roles placed on frontline HCWs over the course of the COVID-19 pandemic, beginning with the risk of COVID-19 infection at work during the initial stages of the pandemic, through to the persistent rise in workplace demands and challenges that exist presently. The perceived impacts of these challenges, including enduring emotional and physical strain, are described by participants.

#### COVID-19 anxiety: lived experiences and impacts

Anxiety in frontline roles during the early stages of the pandemic was a common experience reflected on by many participants. Many felt unprepared and described anxiety provoking experiences as they had little COVID-19 information and working protocols to guide their frontline work. On this experience, participant 8 (F, 38, Nurse, ICU) stated: *“I was extremely anxious during that time*,* not knowing anything and not having access to any information”*. Participants also recalled the challenges with having limited access to personal protective equipment (PPE), as *“all of the PPE got rationed basically immediately”* (M, 27, Nurse, Covid Wards), and working with frequently fluctuating infection control practices, “*it would become an hourly [change] too*,* like ‘oh at [12] o’clock there’s a massive peak in COVID waves’*,* now we’re going to have to go back to wearing the N95 with the shield*,* with the PPE”* (F, 29, Nurse, ICU). Together, these experiences appeared to increase the sense of threat and anxiety amongst staff.

For some, anxiety at work decreased as the pandemic progressed, which participant 8 reported *“It generally improved as it’s gone through now”* (F, 38, Nurse, ICU) and participant 7 said *“I find for me*,* for my skillset now*,* on the floor I’m a lot more confident”* (M,58, Nurse, COVID Ward). Participants attributed this shift in confidence to their increased experience and familiarity with COVID-19-related healthcare work as the pandemic progressed, stating *“my skillset’s better because I’m seeing more and having to do more*,* I’m learning more which I really love. So*,* work wise I think since the pandemic I’m working better*,* and I think that’s because I know I can work at that level”* (M,58, Nurse, COVID Ward). This included training and experience around robust infection control procedures; *“So*,* once you learnt about PPE and proper hygiene and distancing*,* and we did all that every day*,* I don’t think I ever worried about it”* (F, 37, Nurse, Covid Wards).

Participants also noted that during the early stages of the pandemic, they were often worried and hypervigilant about transmitting COVID-19 infections to their family members, expressing concerns for their safety, especially those identified as vulnerable. Participant 1 (F, 64, Nurse, Aged Care) stated that: *“I was concerned about bringing it home because my husband’s got HF COPD”*, and participant 2 (F, 50, Nurse, ED) commented that: *“Everyone was fearful of going home to their families*,* bringing COVID to their families”*. For many, this hypervigilance of infecting family members and concern for their welfare translated into strict personal infection control protocols designed to protect loved ones from possible infection, *“people were showering at work*,* we were taking our clothes of at the front doors*,* when we got home and having a shower before we would let any of our family touch us*,* it was a very fearful sort of time”* (F, 50, Nurse, ED).

Participants reporting ongoing COVID-19 anxiety also noted it was difficult to return to pre-COVID social life despite social distancing restrictions easing, stating that:

*“that’s what I did for many years prior*,* the anxiety that came with having to do that*,* being forced to like isolate*,* …*,* being forced to work during COVID…*,* I guess I shouldn’t say being forced. But it sort of created this whole mindset that…this is what I need to do now. I need to stay here in my little bubble and not contribute to anything else.”* (F,38, Nurse, ED).

#### Relentless demands and fatigue

Many participants described ongoing fatigue and exhaustion, emerging since lockdowns eased. Participants noted that when lockdowns eased, work demands and stressors significantly increased, placing *“lots of pressure on staff”* (F, 56, Allied Health, ICU). Participants’ observations of workplace demands and stressors included the fatigue with wearing PPE when they became available, which many found *“physically very draining”* (F, 56, Allied Health, ICU), and the higher patient acuity when lockdowns eased. They stated that many patients *“were afraid to come to hospital[s] during lockdowns”* (M, 58, Nurse, Covid Ward), resulting in *“a sicker population”* (F, 39, Social Worker, ED), that increased physical and mental demands on staff. Patient care after lockdowns eased was described as “*heavier*,* they’re sicker*,* we’re having to deal with more mental health*,* drug and alcohol*,* eating disorders*,* carers not coping*,* functional declines and all this type of thing that we never did before the pandemic”* (M, 58, Nurse, Covid Ward).

For some, there was added emotional distress and fatigue working alongside the high number of deaths in hospitals during widespread COVID-19 outbreaks, providing a stark contrast to their usual healthcare roles prior to the pandemic. Participants reported “*seeing a lot of the death”* (F, 56, Allied Health, ICU), which was *“quite frightening at times and you felt overwhelmed…even [for] the senior staff”*. Further, *“seeing someone pass on an almost daily basis was [emotionally] taxing”* (F, 37, Nurse, Covid Wards), suggesting healthcare roles were associated with a significant emotional burden.

Participants also reported that their days off and rest were compromised throughout the pandemic, exacerbating fatigue and exhaustion. During periods of lockdown restrictions, participants’ usual opportunities for respite were constrained: *“[I] didn’t feel like there was any down time… There’s no holidays*,* you weren’t able to go anywhere.*” (F, 50, Nurse manager, ED). Social distancing practices also limited respite, *“You weren’t getting that respite and breaks that you would get*,* and you weren’t able to go out and meet with a friend for coffee… so that work-life balance that*,* that connectiveness was difficult”* (F, 37, Nurse, Covid Wards). In the later stages of the pandemic, when staff shortfalls surged, participants reported that guilt with leaving teams understaffed triggered challenges with detaching from work, impacting rest during days off. Participants noted *“even on my days off I felt guilty for not being at work because I knew we were understaffed”* (M, 27, Nurse, Covid Wards) and “*you didn’t feel like you could step away from work and have a normal life*,* so I felt.”* (F, 37, Nurse, Covid Wards).

Most participants reported that they worked overtime throughout the pandemic to compensate for staff shortfalls, compounding their fatigue. Participants frequently worked double shifts, “*some of us were doing night shift as well as day shift*,* it was just relentless”* and in some instances, additional hours beyond that. In Participant 10’s experience,

“*I was on the PM shift when the code blue happened*,* and I then did the double and did the overnight shift as well after that. So*,* the code blue had happened at about six PM that day*,* so three hours into my shift*,* and I then worked additional twelve after it. So just ridiculous number of hours”* (M, 27, Nurse, Covid Wards).

For many, the decision to work overtime and in some instances to forgo leave, stemmed from a sense of team unity coupled with guilt when colleagues were left to work understaffed, stating *“if you knew you were genuinely sick and you needed to call in sick you felt guilty for doing so”* (F, 37, Nurse, Covid Wards). In participant 2’s experience;

*“they’re ringing you*,* can you come in and do a 12-hour instead of 8-hour shift*,* or a double shift. Even though they’re like ‘it’s your choice*,* only do it if you feel you can’*,* not that you feel compelled or responsible for your colleagues*,* but you feel like if there is a possibility that you might be able to help*,* you should do it”* (F, 50, Nurse, ED).

As a result of these experiences, participants described an unending stress associated with their healthcare roles, leading to a *“feeling of helplessness because it was just going on for so long”* (F, 50, Nurse, ED). Participants also reflected on the perceived mental impact resulting from the prolonged fatigue, stating that *“Doing it for two years*,* tired*,* mentally – I think it was just*,* it ended up being the perfect storm for me.”* (M, 58, Nurse, Covid Ward). In Participant 10’s experience, the combination of burnout and managing a code blue incident (cardiac or respiratory arrest in a patient), led to a mental health crisis, *“I ended up on a Workcover claim for that and to*,* I was completely off work*,* not even allowed in the hospital basically for four months. And I was going to weekly therapy sessions and started on a couple of different medications just to help ease some things”* (M, 27, Nurse, COVID Ward). For some, frontline working conditions *“slowly improved”* (F, 37, Nurse, COVID Ward), as improvements in treatment options and workplace support eased some patient care demand. However, many others also noted experiencing an ongoing rise in hospitalisations and workload as outbreaks of COVID-19 infections continues, noting “*the worst is happening now”* (F, 50, Nurse, ED).

### The great resignation

This theme relates globally to the impacts of staff shortages and resignations on frontline healthcare roles observed by participants throughout the pandemic. Participants reflected on the systemic impacts on all levels of staff as resignations surged among senior staff. The ripple effects of these impacts on workplace engagement, connections and quality of patient care were observed and reflected on by participants.

#### Workforce gaps: the impacts and burden on remaining staff

Over the course of the pandemic, participants observed an emerging skills gap due to rising staff resignations as the workforce *“lost so much experience”* (M, 58, Nurse, Covid Ward), specifically senior staff with the experience needed for frontline healthcare work. This was challenging for junior staff to overcome as few senior colleagues remained to meet their support needs. A junior nurse stated: *“we lost some of that support that we were receiving from our educators because we were so short staffed.”* (F, 37, Nurse, Covid Wards) and another nurse redeployed to the ICU noted: *“It was obviously very scary because I don’t have crit care kind of knowledge. I have a very good grasp*,* but still even in the time that I was there*,* very way above my head for a lot of things”* (F, 38, Nurse, ICU). On this issue, remaining senior staff expressed their concerns with the pressure on their inexperienced juniors:

*“They haven’t had that experience and they’re thrown onto wards that are super heavy*,* and people are pushy and you try to support them– all the ones we’ve got they’re great*,* but you can see when you walk onto the ward and the first words that come out is ‘sorry … god*,* it’s been hard’ and that seems to be nearly every day. So*,* it’s just like we’ve hit that pressure and it’s stayed there since the pandemic.”* (M, 58, Nurse, Covid Ward).

As staff resignations rose, participants also described mounting pressure to take on leadership and supporting roles that they felt unprepared for. Participant 10 said “*As somebody who had only graduated in twenty-nineteen it’s not that I wasn’t capable*,* but I was like ‘this is not where I was supposed to be.’ I’m not meant to be senior staff on a ward that I’ve joined three months ago”* (M, 27, Nurse, Covid Wards).

For senior staff taking up these leadership and supporting roles, they reported difficulties with balancing and prioritising supervision of junior staff and their own clinical load. Participants noted that *“There was a lot of pressure on the experienced nurses to not only be looking after your own patients*,* but I had to be aware of what was going on with the other nurses’ patients at the same time. So*,* at any given time I could be responsible for six but pseudo responsible for up to eighteen patients.* (M, 27, Nurse, Covid Wards). As a result, “*that made it hard for [senior staff] to prioritise giving supervision to other staff because client needs often came first*,* patient needs came first.”* (F, 39, Social Worker, ED).

Senior staff noted that balancing these ongoing support needs from junior staff has been stressful and overwhelming, particularly when senior staff themselves require support. Participant 8 stated that: “*I’m making these plans for everyone else. But inside*,* I’m going*,* ‘oh*,* my whole world’s on fire. Help.’ And not putting anything in place for myself either. Yeah*,* it just gets really overwhelming”* (F, 38, Nurse, ICU).

Amidst the rising staff resignations throughout the pandemic, participants also report that the combination of increased workloads and senior staff resignations throughout the pandemic impacted the dynamics, support and morale within healthcare teams for remaining staff, as these workforce issues “*caused a bit of angst amongst the team”* (F, 56, Allied Health, ICU) and *“It was definitely sad seeing a lot of my close friends that I see all the time at work resign”* (F, 29, Nurse, ICU). In a participant’s observation of their workplace:

“*The wards had become during COVID so negative because people were leaving*,* there was no senior staff*,* people didn’t feel supported*,* our ward didn’t have a nurse unit manager”* (F, 38, Nurse, ICU).

#### Disengaging and leaving

Participants reported that frustrations and fatigue associated with senior staff resignations and workforce issues increased disengagement from workplaces, specifically absenteeism among remaining senior staff, stating: “W*e were left with a very sort of junior workforce and those that were senior were just really negative about coming to work*,* a lot of sick leave and all that sort of stuff*,* with nothing there to sort of support them.”* (F, 38, Nurse, ICU).

Participants observed that this disengagement further escalated pressures on remaining senior staff during shifts, leading to a cycle of disengagement and increased strain on them. A senior nurse remarked: “*the pressure was on the senior staff to be able to look after the junior staff*,* so it was a vicious cycle and people did that and didn’t want to come back and so it’s just made this cycle really worse”* (F, 64, Nurse, Aged Care). This negative environment at work also deterred casual staff that were engaged to fill staff shortfalls, *“having to try and support [agency staff]*,* it was mentally tiring and physically tiring. Often [agency staff] not wanting to come back to work because of the poor morale around that”* (F, 37, Nurse, Covid Wards).

Some participants also reported that fatigue and exhaustion has prompted shifts in career plans and intention to change roles in the later stages of the pandemic, stating they *“couldn’t go back into the environment that had been so damaging”* (M, 27, Nurse, Covid Wards). Expanding on this, participant 12 stated *“over the last couple of years I think we’ve*,* I’ve felt like it’s been quite intensive and tiring”*, and as a result *“longer term I may want to change to a community nursing or a GP clinic or something a little bit less physical and less tiring in the future.”* (F, 37, Nurse, Covid Wards).

#### Patient care: the collateral impacts

Due to rising workloads and understaffing, participants observed a decline in the quality of patient care as high number of patients were over allocated to staff, leading to problematic staff to patient ratios. Participants reported “*We went over ratio*,* and we had been over ratio for weeks*” (M, 27, Nurse, Covid Wards), which was frustrating to many as “…*all those things that we fought for as nurses*,* like [staff to patient] ratios and skill mix*,* all went out the window”* (F, 38, Nurse, ICU).

As a result, participants identified challenges maintaining adequate patient care throughout the pandemic, stating *“…it just felt like you physically couldn’t do everything right because there was only one of you and you needed two of you to be able to physically do everything right”* (M, 27, Nurse, Covid Wards). Concerns regarding the implications for patient safety were consequently raised, “*I broke policy because we were under the pump*,* we’re tired*,* we’re just struggling*,* and it happened”* (M, 58, Nurse, Covid Ward).

Some also expressed concerns with recent reduction in PPE resources and shift staffing levels within hospitals when presentations were becoming more severe and complex, *“now they seem to be just pulling back from that and trying to say we can get back to what we used to do*,* but people are sicker today*,* way much sicker today.”* (M, 58, Nurse, Covid Ward). With a lack of resources and staffing, many described feelings of helplessness, noting *“I was feeling helpless or feeling like I wasn’t actually making a difference”* particularly with trying to *“support your patients and your peers.”* (F, 50, Nurse manager, ED).

### Social connections in the COVID-19 era

Within this theme, participants described their experiences throughout the COVID-19 pandemic related to social connections with the community, their families and in their workplaces as frontline HCWs. These experiences revolved around tensions with the community, as well as the challenges with managing work life balance as frontline HCWs. Participants also reflected on the support workplace relationships provided them throughout the pandemic.

#### Community disconnectedness

Participants reflected on living and working in a community that had opposing views and practices to theirs, voicing their frustrations with increasingly abusive patients throughout the pandemic. Participant 3 stated that:

*“being real anti-vaxxers and being really resistant to treatment even though they were there in hospital; they were argumentative; they weren’t intubated;… they were resistant; they were sometimes quite aggressive*,* and that created actually a huge amount of stress on the likes of me and the medical team*,* like all of us. It was something that we had not really… encountered before”.* (F, 56, Allied Health, ICU)

Participants also observed a disconnect with their community, given the dissonance in community infection control practices and the rigorous standards required of healthcare workers within hospitals throughout the pandemic. Such discrepancies were often perceived by participants as a slight to their extensive efforts to safeguard their community.

*“you’re put in a situation where you’re working 12 plus hours a day and trying to keep people safe and then you’re back out into the community*,* and it’s ‘we shouldn’t be wearing masks’ – that just infuriates me. Because they’re not – and they’re not seeing what we are doing in the hospitals*,* day after day after day*,* to try and keep as many people as safe as possible.”* (F, 50, Nurse manager, ED).

Participants also described feelings of disconnection from their community who were moving past the worst of the pandemic, while they continued to work in healthcare roles under strict COVID-19 conditions, stating : “*it’s just frustrating because it felt like sometimes people were sort of moving on with their lives*,* however we were sort of still stuck with the pandemic regulations that the hospital put on*,* [to] protect ourselves and the patients”* (F, 29, Nurse, ICU).

#### Work to family spillover effects: impacts on connections and cohesion

Participants noted that the heightened workplace demands over the course of the pandemic had a negative impact on family life. For some participants, overtime and filling in shortfalls due to staff shortages led to family time being sacrificed. Participant 6 noted, “*worked too many hours if I’m honest and look[ing] back at that*,* sacrificed a lot of family time”* as she “*often miss out in my family things like my children’s sport games or family events”* (F, 39, Social Worker, ED).

Participants also recalled isolating themselves from their family during the pandemic, as many were hesitant to affect family members with traumatic work-related stress, stating *“you don’t want to talk about the horrible way that somebody died in front of you all that time. So*,* I would come home from work and just isolate myself and try to keep it all in and not talk about it”* (M, 27, Nurse, Covid Wards). When there were mental health concerns among family members, this isolation was further exacerbated. Participant 8 reported: *“One of [my daughters] got quite severe anxiety and [she] would freak out if anyone so much as got*,* you know*,* in the same room as her as it was. So*,* [I was] sort of isolating everything that was going on at work just to myself*,* and not sharing it with anyone”* (F, 38, Nurse, ICU).

In some instances, participants noted that work related stress during the pandemic affected family relationships, resulting in *“breaks down in relationships”* (F, 38, Nurse, ICU). For some participants, this was a result of work-related stress inadvertently aggravating their behaviour at home:

*“My husband tells me now that I was a menace to be around at home*,* that I was on a hairpin trigger and just honestly a bit of a [@#!$] to live with in all seriousness. I thought*,* I was trying really-really hard not to*,* because what I was going through at work was very traumatic*,* I knew it was very traumatic*,* and I also knew that it wasn’t fair to put that on the people that I lived with*” (M, 27, Nurse, Covid Wards).

#### The supportive role of workplace relationships

For participants experiencing role-related stress, workplace relationships were frequently cited as important source of support, as colleagues were able to rapidly empathise and relate to their situation:

“*if I had issues I could just go and talk to my friends about it*,* and they’d understand it. I don’t have to like*,* go in*,* explain the situation*,* I could just turn to my friend*,* tell them what’s happening*,* then it sort of just unloads a lot of my anxiety or sadness or something and then I feel heaps better”* (F, 29, Nurse, ICU).

In most cases, these collegial interactions occurred in informal settings where participants reported that healthcare teams debriefed and supported each other openly. Participant 7 reported that, *“we’ll go out for dinner*,* drinks – I call it a debrief”*, and in these informal gatherings peers were consistently supportive of each other; “*It was like it was the R U OK program every day for 18 months type of thing*,* making sure everybody was okay”* (M, 58, Nurse, Covid Ward).

More formal team meetings in the workplace were also reported by participants to help with mitigating uncertainty in frontline roles throughout the pandemic, as it helped healthcare teams with *“knowing what was going on”* (F, 64, Nurse, Aged Care). During these team meetings, participants emphasised the importance of leaders openly communicating organisational changes and updates, “*someone who goes to the team meetings*,* to [give] that information about what’s happening with the organisation*,* about what’s opening and what’s not”* (F, 38, Nurse, ICU).

Formal team debriefs were also noted to play a role in mitigating the sense of unpreparedness felt by staff as it helped improve and adapt clinical practices that were highly dynamic throughout the pandemic, citing it was essential “*for a collective team to be able to discuss the challenges that we were facing*,* and what we were learning*,* and what worked well*,* and what didn’t work well”* (F, 37, Nurse, Covid Wards).

Participants also described the role positive and supportive supervisory relationships played in mitigating distress throughout the pandemic. They particularly valued open and trusting relationships that made supervisors more approachable when seeking support. Participant 8, who reported having a supportive supervisor, valued “*being able to have that interaction with her*,* being able to know if something was wrong I could always go to her*,* just being really open*,* door always open.”*. Participants also felt supported when their difficulties were acknowledged, “*just going*,* ‘we see these issues and we are trying to do this’*,* and ‘we understand how hard that [it is]’”* (F, 38, Nurse, ICU). This included feeling supported when there was acknowledgement of the many family challenges participants faced throughout the pandemic: *“acknowledging that it would be very hard with some people out in their family life struggling with things”* (F, 56, Allied Health, ICU).

Conversely, distress intensified when supervisors were unreceptive and neglected staff’s concerns, as observed in participant 2’s experience:

feeling of not being heard, not being listened to, not being allowed to collaborate. And this was as a result of the person that was actually leading the team at the time…– and I stormed out and burst into tears and I was just – this is just ridiculous.

## Discussion

This study explored HCWs’ experiences of frontline roles throughout the last three years of the COVID-19 pandemic, with a particular focus on the workplace, relational health, mental health and wellbeing. Anxiety was a prominent theme discussed, initially driven by uncertainty in frontline healthcare roles, lack of COVID-19 information, and safety of family members. Late stage COVID-19 anxiety was also discussed, which centred around community infections and social interactions. Significant increases in staff resignations were reported, placing unprecedented pressure on remaining staff who described a decline in their mental health and wellbeing as a result. The findings also offer insights into the social support needs and opportunities available for frontline HCWs, offering evidence for governments, health organisations and healthcare leaders when developing future supports and strategies, particularly in the context of ongoing challenges for the sector.

### Outbreak anxiety

Anxiety among frontline HCWs has been a particularly salient issue since the beginning of the COVID-19 pandemic as studies have shown that working in high-risk settings during the pandemic has led to significantly greater risk of experiencing anxiety [9]. Our study provides further insight, demonstrating the changing nature of frontline HCWs’ anxiety through the course of the pandemic.

Frontline HCWs in our study identified pandemic unpreparedness, insufficient protection, and fluctuating infection control practices in the initial stages as workplace stressors that were anxiety provoking. This is consistent with research from the COVID-19 pandemic [25, 47, 48] and previous coronavirus outbreaks [11, 49, 50], suggesting that inadequate safety for staff, uncertainty in frontline roles and insecure workplace practices during infectious disease outbreaks can be detrimental to staff mental health and wellbeing, particularly if it revolves around clinical practices and organisational communication. The findings here reinforces studies that have shown that unpredictability in implementing new workplace practices and organisational changes, which are inherent in infectious disease outbreaks, can lead to emergence of unforeseen stressors that can pose significant risks to mental health and wellbeing [51–53].

Our findings also suggest that more needs to be done to understand prolonged COVID-19 anxiety among frontline HCWs. Our study found that there was sustained hypervigilance of COVID-19 infections that continued to negatively influence social behaviours among some frontline HCWs at the end of the pandemic. This is concerning, as our findings, along with others [38], observed extensive hypervigilance of threat among frontline HCWs that began in the initial phase of the pandemic, over three years ago. This initial hypervigilance was centred around the potential threat they posed to their families as carriers and vectors of infection. While this extensive hypervigilance and attentional bias to threat among frontline HCWs is a typical behavioural response that can act as a protective mechanism [54], and is arguably supportive of infection control practices, it has been widely evidenced to be associated with the aetiology and maintenance of anxiety disorders [55–57]. Emerging research from the COVID-19 pandemic supports this contention, suggesting that prolonged attention to COVID-19 threat can exacerbate and maintain anxiety [58–60]. Though more research is needed to further understand the influence of attentional bias to COVID-19 threat in frontline HCWs’ anxieties at work, these findings nonetheless provide insights into the likely psychological mechanisms involved in prolonged COVID-19 anxieties among frontline HCWs, which can better inform mental health support practices and mental health treatment targets for this cohort.

***Policy implications.*** Collectively these findings underscore the importance of workplace safety and infection control practices during infectious disease outbreaks. Based on the findings, it is recommended that at the government and organisational level effective communications is prioritised in their crisis response during infectious disease outbreaks, to mitigate the impacts of uncertainty and unpredictability during crisis phases of an infectious disease outbreak. Specifically, prioritising clear, iterative, and a direct line of communication; one that meets the changing needs of frontline HCWs during crisis phases of infectious disease outbreak [25]. Additionally, the findings highlight the need for healthcare leaders to recognise and address poor staff wellbeing beyond the COVID-19 pandemic, including prolonged COVID-19 anxiety and distress among staff, which has significant implications for both frontline HCWs’ mental health and the financial burden on organisations. It was estimated that psychological distress potentially cost Victorian (Australia) organisations from $859 to $1461 AUD per frontline staff per month during the COVID-19 Omicron wave [61], which would equate to $171 800 to $292 200 for a major Victorian (Australia) emergency department with 200 staff [62]. There is thus significant value in organisational strategies and support that address the potential of prolonged COVID-19 anxiety and distress among frontline HCWs, such as early identification of mental health issues [63], improving help-seeking behaviour [64], and the use of tailored psychological interventions [65]. Professional practice guidelines for employee assistance programs and mental health practitioners based on this research have also been developed specifically for frontline HCWs that can assist mental health practices when addressing these issues among this cohort [66].

### Workplace psychosocial demands

Concerns over staff shortages in the healthcare sector and its impacts on frontline HCWs have emerged years before the COVID-19 pandemic. However, our study shows that the COVID-19 pandemic has been a catalyst for further resignations and poor staff engagement, contributing to a rise in workplace psychosocial demands for the cohort. Frontline HCWs identified mental and physical challenges with the need to balance the increase in physical workloads, support for junior staff, specialised work, and the rapid uptake of leadership roles; all while they have little and compromised respite. The impacts of these demands are wide ranging. Research supports that work-related strain during the COVID-19 pandemic left frontline HCWs vulnerable to increased psychological distress [67, 68]. Furthermore, our study has observed a recursive and circular loop, where staff resignations and shortages trigger an escalation in demand that increase stress and fatigue, and subsequently more absenteeism and intention to leave among staff. Other studies have observed similar outcomes, providing further evidence that unmanageable increases in demands will exacerbate disengagement [69] and turnover intention among frontline HCWs [70]. Our findings highlight a growing dilemma for healthcare organisations, especially if staffing issues are not addressed.

***Policy implications.*** With staff shortages continuing to rise globally [71], it is critical that Australian healthcare organisations innovate support and incentives to recruit and retain Australian staff to address the overwhelming demands on the healthcare workforce. While financial incentives remain an effective strategy, our study highlights the critical need to improve workplace conditions to alleviate demands and mitigate its impacts, which is important to ensure the healthcare sector remains an attractive place to work. On improving workplace conditions, Maben et al. [72] suggests that more needs to be done to improve workplace culture, staffing ratios and psychological safety; which is dependent on organisational leadership, support management and role modelling.

Organisational strategies need to also consider the importance of mental health and wellbeing in attracting and retaining staff. An important step is to reduce and mitigate the mental health and wellbeing impacts through organisational strategies mentioned above, however, given the extent of staff shortages and resignations, strategies aimed at attracting and retaining staff need to go beyond. To attract and retain the incoming generation of healthcare staff, organisational strategies need to also consider the significant value in fostering and cultivating positive mental health and wellbeing in healthcare workplaces [73], using novel strategies. Studies suggest that leveraging extrinsic rewards can be beneficial in attraction strategies (e.g., wellbeing allowances, workplace benefits, financial incentives, salary increases), while intrinsic rewards is important for retention strategies (e.g., public service motivation, career growth, mentoring opportunities, employee autonomy) [74].

This study also suggests that to strengthen workforce retention and sustainability, there needs to be immediate and substantial increases in workplace resources, support, and training for supervisors. Effective organisational support for supervisors is critical given the rapid uptake of leadership roles in the recent years and the impacts this have on their capacity and capabilities to subsequently provide essential support to their staff and sustain healthcare teams. Furthermore, based on the job demands-resource model, our study also suggests that organisations can mitigate work-related strain and reduce mental health impacts through policies that aim to improve and strengthen social support for their staff [75].

### Social support

Research on social support in the healthcare workplace, both before [76, 77] and during the COVID-19 pandemic [78–81], have shown that collegial support is a key influencing factor in frontline HCWs’ mental health. In keeping with this large body of evidence, our study identified that collegial support within their teams were valuable resources in mitigating work-related strain and mental health impacts throughout the COVID-19 pandemic. Our study, along with [82], suggests that frontline HCWs value collegial supports in informal settings as forms of rapid debriefing with someone who had shared experiences of their issues. It seems that frontline HCWs perceive their issues to be uniquely traumatic, making it more appealing to engage with colleagues for mental health and wellbeing support, as they can understand them quickly and provide them with more relatable supports. However, it also appears that these strong informal relationships have made it difficult for frontline HCWs to set boundaries within their collegial relationships. Our findings showed that many frontline HCWs experienced guilt when taking leave, as many put significant emphasis on supporting each other, putting their colleague’s wellbeing before themselves. While overall these findings affirms the use of formal peer support interventions in healthcare settings [83–85], it also highlights organisations’ crucial role in enhancing and promoting healthy workplace relationships, as well as providing frontline HCWs with the tools and resources, such as workplace mental health training, to support each other organically and effectively in informal settings.

Related literature underscores the value and benefits of supervisor support to improve frontline HCWs’ mental health and wellbeing [10, 86, 87]. This is an important finding given that studies have found that supportive supervisors can mitigate the concerning levels of turnover intention and disengagement among the healthcare workforce [88, 89].This study further reveals that frontline HCWs particularly value open and transparent communication from supervisors; an important relational component that studies have shown fosters a sense of trust in the healthcare workplace, and enhance workplace relationships between supervisors and staff, as well as within teams [90–92]. This is of significance, as open communication and the sense of trust can foster openness between staff to share ideas and issues, such as mental health problems, promoting better help-seeking behaviour among a traditionally reticent workforce [64].

Given these findings, it is evident that social support is an effective and preferred resource for frontline HCWs to cope with mental health challenges. However, our findings also indicate that staffing issues and shortages may be eroding these resources. Our study has for the first time shown that staff shortages and workforce issues have not only increased demands, but also impacted the social environment of frontline HCWs, which had subsequent implications on staff engagement and absenteeism. Our participants reported that due to rising staff resignations, there has been more aversive social interactions at work and loss of important collegial relationships that were crucial to their support systems. Furthermore, supporting previous findings [93–95], our study showed increasing experiences of work-family conflict, which is not only detrimental to frontline HCWs’ social support systems, but also has significant implications on reducing job satisfaction [96] and further increasing turnover intention [97]. These social support systems are crucial to frontline HCWs, as studies have shown that frontline HCWs rarely use and rely on formal psychological supports despite having access and knowledge of them in the workplace [98, 99]. Social support is thus heavily relied on by frontline HCWs to cope with mental health challenges. As a result, if social connections continue to deteriorate, frontline HCWs will be left with a dearth of mental health support, resorting to maladaptive coping strategies, such as alcohol use, which has been on the rise among HCWs [9, 100–102]. It will also contribute further to the rise in intention to leave and staff shortages in the healthcare workforce [103, 104]; maintaining the overwhelming demands and further deteriorating workplace social connections. These findings therefore underscore the critical need to not only develop social interventions, but also protect and enhance frontline HCWs social supports now and in future infectious disease outbreaks.

***Policy implications.*** Workplace relationships are an essential component to a positive workplace culture and climate, and thus should be a priority in organisational strategies and social interventions to reduce workplace distress and improve positive mental health and wellbeing among staff. The findings in this study indicate that key organisational actions to achieve these goals would include improving on-boarding for young and new staff, fostering positive team relationships, and improving work-family balance. It is also recommended that supervisors are leveraged within these strategies and interventions, as they have been found to be the initial point of contact for support when stressors arise [105], presenting a critical opportunity for targeted intervention. Their role in shaping healthcare workers’ mental health is now well-established, with consistent recognition of the need to enhance supervisory support for HCWs [80, 106–108], underscoring a key area for change within the healthcare sector.

Existing interventions that can be implemented at the supervisor level include training on family-supportive supervisor behaviors [109] and building skills in communicating and providing practical mental health support for staff [110, 111]. Adding to these interventions, this study recommends that supervisory training should also include a focus on building their capacity and capabilities in (1) fostering more open relationships with staff, (2) improving staff’s help-seeking behaviours, and (3) cultivating positive workplace culture and team dynamics. However, it is important to note that these organisational strategies aimed at improving supervisory support should be cautious and strategic, ensuring that supervisors are given the proper resources to support staff, as it has been shown that increased support responsibilities can elevate workplace demands and place additional burden on supervisors [112].

### Strengths and limitations

A strength in our study is the diverse team that has brought multiple perspectives to the data analysis, allowing for an in-depth and rich data analysis into the experiences and themes emerging from interviews. This is an important component for a qualitative analysis to capture a comprehensive picture of participants experiences, without losing individual and important themes. When considering the findings, however, it is important to note the limitations of the study. Specifically, despite our study’s intent to capture a diverse sample, the study lacked doctors in the sample, which was a challenge to recruit due to their busy workloads. Although the findings may apply to their cohort, there may be a gap in unique experiences and themes related to doctors’ frontline roles. Future research should consider innovative approaches to recruit this sample that includes incentives and benefits to this busy cohort. Additionally, given the retrospective and qualitative nature of the research, recall bias is inherent within the study design and there may be gaps in the reports of individual participants, particularly with their experiences early in the COVID-19 pandemic. Nevertheless, although recall bias is an inherent limitation of this retrospective qualitative design, the consistency of themes across individual participants and the persistence of participants’ recollections over time suggest that these experiences were particularly salient, offering essential insights into the key experiences and enduring needs of healthcare workers throughout the pandemic. Lastly, the qualitative approach used in this study limits the exploration of relationships between themes. Future research is required to examine these relationships using more robust quantitative methodologies.

## Conclusion

Despite the declaration of the end of the COVID-19 pandemic, COVID-19 outbreaks persist in many countries and continue to affect frontline HCWs mental health and wellbeing. While acute COVID-19 infection risks have subsided, staff shortages have surged, leading to incredible workforce issues and impacts on frontline HCWs’ social support systems, mental health, and wellbeing. The issues here, if they persist, will lead to detrimental impacts on staff recruitment and retainment, as healthcare roles will no longer be an attractive career to the incoming generation of workforce. It is thus critical that the next set of strategies and further research in the healthcare sector addresses these workforce issues, as well as further the understanding of frontline HCWs mental health to enhance and support more tailored and consumer-led solutions and interventions that is focused on effectiveness and relevance for this unique cohort.

## Supplementary Information

Below is the link to the electronic supplementary material.

Supplementary Material 1

Supplementary Material 2

## Data Availability

The datasets used and/or analysed during the current study are available from the corresponding author on reasonable request.
