# Supplementary material for: Three years of pandemic stress and staffing challenges: a retrospective qualitative study of COVID-19 impacts on frontline healthcare workers’ mental health and wellbeing
Source: BMC Psychiatry. 2025 Oct 30;25:1036. doi: 10.1186/s12888-025-07348-4 (PMC12573931; doi:10.1186/s12888-025-07348-4)
Supplement: Supplementary file 1 — Supplementary Material 1 [file 12888_2025_7348_MOESM1_ESM.docx]

| **No** | **Item** | **Guide questions/description** | **Article Page Number/response** |
| --- | --- | --- | --- |
| **Domain 1: Research team and reflexivity** | | | |
| Personal Characteristics | | | |
| 1 | Interviewer/facilitator | Which author/s conducted the interview or focus group? | Reported in page 3 |
| 2 | Credentials | What were the researcher's credentials? E.g. PhD, MD | Reported in page 5 |
| 3 | Occupation | What was their occupation at the time of the study? | Reported in page 5 |
| 4 | Gender | Was the researcher male or female? | Male and Female |
| 5 | Experience and training | What experience or training did the researcher have? | BL, JS, and LB have extensive experience and training with qualitative research and training, with a wide range of publications and qualitative research in healthcare research. ML and CO have extensive experience in health research, particularly in the field of developmental psychology. HLH is an emerging qualitative researcher in psychology. |
| Relationship with participants | | | |
| 6 | Relationship established | Was a relationship established prior to study commencement? | No |
| 7 | Participant knowledge of the interviewer | What did the participants know about the researcher? E.g. personal goals, reasons for doing the research. | Participants had no existing knowledge or relationship with the interviewers (BL and HLH) |
| 8 | Interviewer characteristics | What characteristics were reported about the interviewer/facilitator? E.g. Bias, assumptions, reasons and interests in the research topic | Reported in page 5 |
| **Domain 2: study design** | | | |
| Theoretical framework | | | |
| 9 | Methodological orientation and  Theory | What methodological orientation was stated to underpin the study? E.g. grounded theory, discourse analysis, ethnography, phenomenology, content analysis | Reported in page 4 |
| Participant selection | | | |
| 10 | Sampling | How were participants selected? e.g. purposive, convenience, consecutive, snowball | Reported in page 4 |
| 11 | Method of approach | How were participants approached? e.g. face-to-face, telephone, mail, email | Reported in page 4 |
| 12 | Sample size | How many participants were in the study? | Reported in page 4 |
| 13 | Non-participation | How many people refused to participate or dropped out? Reasons? | None |
| Setting | | | |
| 14 | Setting of data collection | Where was the data collected? e.g. home, clinic, workplace | Reported in page 4 |
| 15 | Presence of non-participants | Was anyone else present besides the participants and researchers? | No |
| 16 | Description of sample | What are the important characteristics of the sample? e.g. demographic data, date | Reported in page 4 and 5 |
| Data collection | | | |
| 17 | Interview guide | Were questions, prompts, guides provided by the authors? Was it pilot tested? | Appendix A |
| 18 | Repeat interviews | Were repeat interviews carried out? If yes , how many? | No |
| 19 | Audio/visual recording | Did the research use audio or visual recording to collect the data? | Both |
| 20 | Field notes | Were field notes made during and/or after the interview or focus group? | Both |
| 21 | Duration | What was the duration of the interviews or focus group? | Reported in page 4 |
| 22 | Data saturation | Was data saturation discussed? | Yes |
| 23 | Transcripts returned | Were transcripts returned to participants for comment and/or correction? | Reported in page 4 |
| **Domain 3: analysis and findings** | | | |
| Data analysis | | | |
| 24 | Number of data coders | How many data coders coded the data? | one |
| 25 | Description of the coding tree | Did authors provide a description of the coding tree? | Reported in page 4 |
| 26 | Derivation of themes | Were themes identified in advance or derived from the data? | Derived |
| 27 | Software | Were software | Reported in page 4 |
| 28 | Participant checking | Did participants provide feedback on the findings? | Reported in page 4 |
| Reporting | | | |
| 29 | Quotations presented | Were participant quotations presented to illustrate the themes / findings? Was each quotation identified? e.g. participant number | Yes, reported in throughout the results |
| 30 | Data and findings consistent | Was there consistency between the data presented and the findings? | Yes, reported in throughout the results |
| 31 | Clarity of major themes | Were major themes clearly presented in the findings? | Yes, reported in throughout the results |
| 32 | Clarity of minor themes | Is there a description of diverse cases or discussion of minor themes? | Yes, reported in throughout the results |
