# Supplementary material for: Three years of pandemic stress and staffing challenges: a retrospective qualitative study of COVID-19 impacts on frontline healthcare workers’ mental health and wellbeing
Source: BMC Psychiatry. 2025 Oct 30;25:1036. doi: 10.1186/s12888-025-07348-4 (PMC12573931; doi:10.1186/s12888-025-07348-4)
Supplement: Supplementary file 2 — Supplementary Material 2 [file 12888_2025_7348_MOESM2_ESM.docx]

# COVID-19 and FHWs: Adult Interview Questions

*Date: November 2022*

*Full Project Title: Investigating the impact of the COVID-19 pandemic on Victorian healthcare workers.*

# SECTION 1: PREAMBLE

Thank you for agreeing to participate in this interview.

The aim of this interview is to gain an understanding of the continuing impact of frontline health work during the COVID-19 pandemic on your mental health and wellbeing.

I just want to re-iterate that as always, your participation in this research is entirely your choice. Also, it is entirely up to you whether you want to answer all of my questions. So, if there’s a question that you’d rather not answer, you can just let me know and we’ll move straight on to the next question

All the data that I collect from you will be treated in a confidential manner, so I’ll be removing all information that identifies you, and you will again have an opportunity to check that yourself because you’ll be receiving a copy of the transcript before I do any analysis.

I also want to remind you that these interviews are confidential, what you say today won’t be shared with = your workplace and we will not include any personal information in our results. The only exception to this will be if we have any concerns about the safety of yourself or someone else. With that in mind, we would also ask that your answers to these questions are focused on your experiences with working in healthcare during the pandemic rather than personal details about individual family members, workplaces, or colleagues.

Even though I understand that you have provided consent to having this interview being taped, I just need to confirm again that you are okay with having the interview recorded? (If yes, start recording)

# Healthcare worker specific questions

- **How would you describe your overall wellbeing throughout the pandemic?**
  - **Prompt:**
    - **For mental wellbeing**
    - **For different stages of the pandemic, e.g., the start of the pandemic.**
- **What were the most significant challenges you faced during the pandemic?**
- **What impact(s) did they have on your mental health and wellbeing? (prompts to consider:**
  - **Prompt:**
    - **stigma in community or extended family, workload, anxiety re: risks, relationship stress, lockdowns, lack of experience**
    - **Challenges faced as a community member, frontline Healthcare Worker, a young adult/new workforce (if relevant)**
- **During this time, what informal strategies, tools or supports did you use to cope with the challenges?**
- **During this time, what formal strategies, tools or supports did you use to cope with the challenges?**
  - **Can you tell me more about your experiences with these strategies/tools/supports**
    - **Prompt:**
      - **Are you still using them?**
      - **What do you like about them?**
      - **What do you dislike about them?**
      - **What prompted you to engage them?**
      - **Have you accessed formal supports or interventions for mental health (EAP, psychologist, GP)? What have your experience with them been like?**
  - **For those that cannot identify formal or informal supports: can you tell me about the possible barriers that may have impacted your access?**
- **How would you assess your work-life balance prior to the pandemic? How did it change through the course of the pandemic?**
- **Ho*w* have you managed the demands associated with frontline healthcare work through the pandemic?**
  - - **Have you observed anything to be helpful in managing the demands?**
    - **Unhelpful?**
- **What role, if any, do you think your workplace have in helping staff to manage the challenges/demands you have experienced during the pandemic?**
  - **Prompt:**
    - **What is your experience of workplace support during this time?**
      - **Prompt for supervisor support, organisational support**
    - **In terms of your healthcare role, do you think your workplace is psychological safe (i.e., appropriate work demands, supportive environment, control over work/role) for you? Can you tell me more about this?**
    - **What, if anything, could have been done to better support you?**
- **Have your long-term career interests/focus changed as a result the pandemic?**
  - **In what ways?**
  - **What do you think contributes the most to these changes in your thoughts? (pandemic, organisation, systemic impacts, family impacts)?**
- **Do you have any additional comments or observations about your role as a frontline healthcare worker during the pandemic?**

**CONCLUSION**

That is all the questions I have for you. Are there any other comments, issues or concerns you would like to make about this?

I will be sending you a transcript of this interview for you to check and delete any information you think might be potentially identifiable or to add anything you might like to add. You will have two weeks to do this but if you need more time please let me know. What is the best way for me to get this to you?

Thank you so much for your time. We appreciate it. I need to remind you that if you find this process distressing either now or later there are people to talk to. Just let me know and I can arrange this for you.
